# Activation of JNK Signaling Mediates Connective Tissue Growth Factor Expression and Scar Formation in Corneal Wound Healing

**DOI:** 10.1371/journal.pone.0032128

**Published:** 2012-02-21

**Authors:** Long Shi, Yuan Chang, Yongmei Yang, Ying Zhang, Fu-Shin X. Yu, Xinyi Wu

**Affiliations:** 1 Department of Ophthalmology, Qilu Hospital, Shandong University, Jinan, People's Republic of China; 2 Departments of Ophthalmology, Anatomy, and Cell Biology, Wayne State University School of Medicine, Detroit, United States of America; Institut national de la santé et de la recherche médicale - Institut Cochin, France

## Abstract

Connective Tissue Growth Factor (CTGF) and Transforming growth factor-β1 (TGF-β1) are key growth factors in regulating corneal scarring. Although CTGF was induced by TGF-β1 and mediated many of fibroproliferative effects of TGF-β1, the signaling pathway for CTGF production in corneal scarring remains to be clarified. In the present study, we firstly investigated the effects of c-Jun N-terminal kinase (JNK) on CTGF expression induce by TGF-β1 in Telomerase-immortalized human cornea stroma fibroblasts (THSF). Then, we created penetrating corneal wound model and determined the effect of JNK in the pathogenesis of corneal scarring. TGF-β1 activated MAPK pathways in THSF cells. JNK inhibitor significantly inhibited CTGF, fibronectin and collagen I expression induced by TGF-β1 in THSF. In corneal wound healing, the JNK inhibitor significantly inhibited CTGF expression, markedly improved the architecture of corneal stroma and reduced corneal scar formation, but did not have a measurable impact on corneal wound healing in vivo. Our results indicate that JNK mediates the expression of CTGF and corneal scarring in corneal wound healing, and might be considered as specific targets of drug therapy for corneal scarring.

## Introduction

The cornea is a highly transparent tissue located at the anterior surface of the eye. Corneal scarring caused by injury or surgery is one of the main causes of blindness worldwide [1]. So far, there is no effective and safe strategy for the prevention or inhibition of corneal scar formation in clinical practice. Therefore, research on how to reduce corneal scarring in corneal wound healing will be of great clinical value.

TGF-β1 has been found to play an important role in promoting fibrosis and scarring in numerous tissues [2]. Many of the scarring effects of TGF-β1 are mediated by CTGF [3]. CTGF is a 38-kDa secreted protein belonging to the CCN family [4], and its expression is induced by TGF-β1 in cultured fibroblasts [5], [6]. CTGF has been shown to promote the synthesis of various constituents of the extracellular matrix [7], [8] and its over-expression can promote fibrosis and scar formation in skin, kidney, liver, brain, lung, human gingiva, vasculature and pancreas [9], [10], [11].

TGF-β1 and CTGF are key growth factors in regulating corneal scarring [12], [13]. We have previously shown that expression of TGF-β1 and CTGF increased dramatically during corneal wound healing, TGF-β1 could induce CTGF expression in vivo [14]. TGF-β1 played an important role in the activation of quiescent corneal keratocytes [15], CTGF was induced by TGF-β1 and mediated the effect of TGF-β1 on collagen, fibronectin synthesis [16]. This was consistent with other reports in which TGF-β1 increased CTGF expression in human corneal fibroblasts [12]. Antisense oligonucleotides and neutralizing antibodies to CTGF decrease TGF-β1 induced collagen synthesis, cell proliferation and matrix contraction in corneal fibroblast [17], [18]. CTGF plays a critical role in mediating many of the important fibroproliferative effects of TGF-β1 in corneal fibroblasts. Therefore, understanding mechanisms regulating expression of CTGF increased by TGF-β1 is of great importance to inhibit corneal scarring.

SMAD proteins are the primary substrates of TGF-β1 receptors [19], whereas we previously found that TGF-β1 up-regulated CTGF expression was not via SMAD pathways in rabbit corneal wound healing [14]. In addition to SMAD proteins, the mitogen-activated protein kinase (MAPK) pathways were involved in TGF-β1 signaling [20]. MAPK pathways are a family of serine-threonine protein kinases that are activated in response to a variety of extra cellular stimuli. Extracellular signal-regulated kinase (ERK), JNK and p38 pathway constitute three major subfamilies of MAPK pathways [21]. It has been shown that TGF-β1 can activate the ERK [22], JNK [23] and p38 [24] pathway. There is evidence that TGF-β1 induced CTGF expression is mediated through JNK in human lung fibroblasts [25]. In gingival fibroblasts, the sole MAPK mediates the TGF-β1 stimulated CTGF expression was JNK [26]. ERK mediates TGF-β1 induced CTGF expression in skin fibroblasts [27]. Inhibition of p38 could suppress collagen I, fibronectin and CTGF expression induced by TGF-β1 in conjunctival fibroblasts [28]. Our Previous studies have shown that TGF-β1 induced the activation of JNK in corneal fibroblast, inhibition of JNK pathway can effectively inhibit TGF-β1 induced CTGF expression and subsequent corneal fibroblast proliferation and collagen over-expression in corneal fibroblasts [15]. However, the signaling pathway of CTGF production in corneal wound healing remains unclear.

Based on these findings, it was hypothesized that MAPK pathways could mediate CTGF expression and corneal scarring in corneal wound healing. In the present study, we investigated whether TGF-β1 could induced MAPK pathways phosphorylation in THSF cells, and determined the effect of the MAPK pathways in TGF-β1 induced CTGF, fibronectin and collagen I mRNA expression in THSF cells were investigated. Then, the penetrating corneal wound model was created in vivo and the effect of JNK on CTGF expression and corneal scarring in corneal wound healing was identified.

## Results

### TGF-β1 induced MAPK pathways phosphorylation in THSF cells

**Figure 1:**
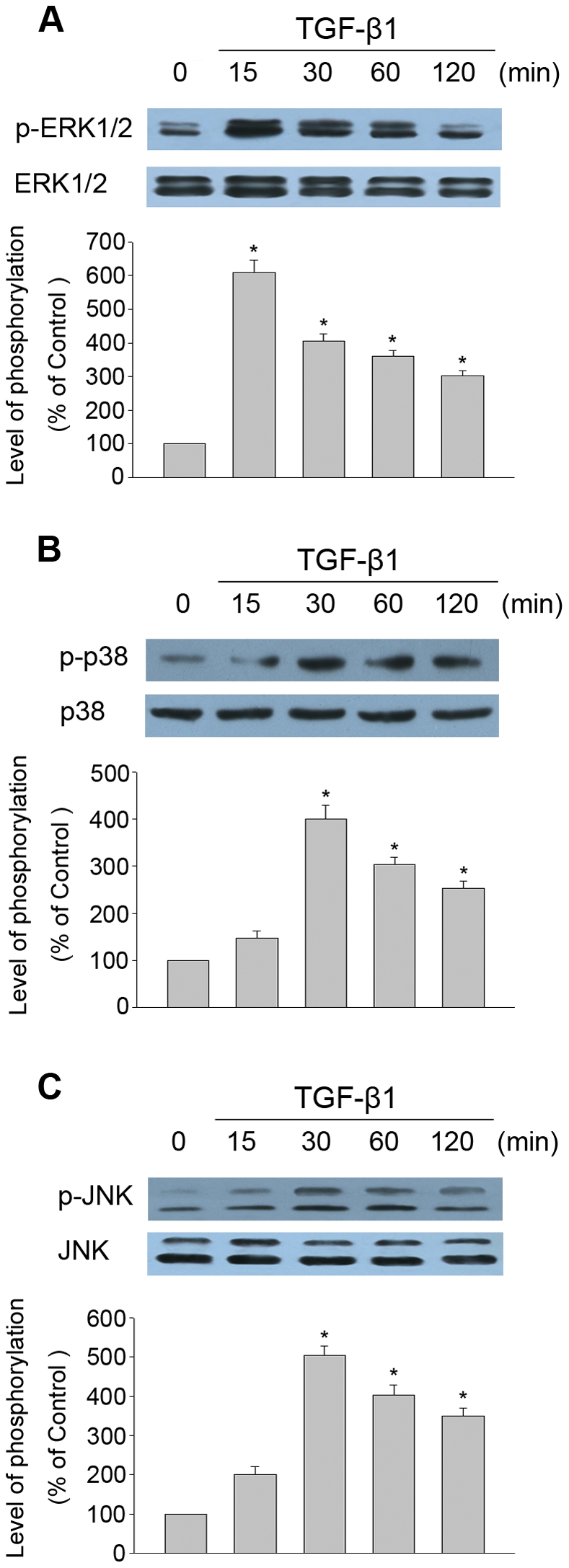
TGF-β1 induced MAPK pathways phosphorylation in THSF cells. THSF cells were incubated with TGF-β1 (3 ng/ml) for the times indicated. The total and phosphorylation of ERK (A), p38 (B) and JNK (C) MAPK were determined by using Western blot analysis. Data are representative of three independent experiments. *, P<0.05 vs. control cells without TGF-β1 stimulation.

We investigated whether TGF-β1 could induce MAPK pathways phosphorylation in THSF cells. THSF cells were treated with 3 ng/ml of TGF-β1 for 15, 30, 60 and 120 minutes, followed by extraction of the cellular protein. The expressions of total and phosphorylated ERK1/2, p38, and JNK were determined by Western blot analysis. As shown in Figure 1, THSF cells stimulated with TGF-β1 induced a rapid increase in the phosphorylation of ERK, p38 and JNK. The maximum phosphorylation of ERK was observed after 15 min of stimulation with TGF-β1. While the maximum phosphorylation of p38 and JNK were observed after 30 min of stimulation with TGF-β1.

### Inhibitory effect of PD98059, SB203580 and SP600125 on TGF-β1 induced MAPK pathways phosphorylation

**Figure 2:**
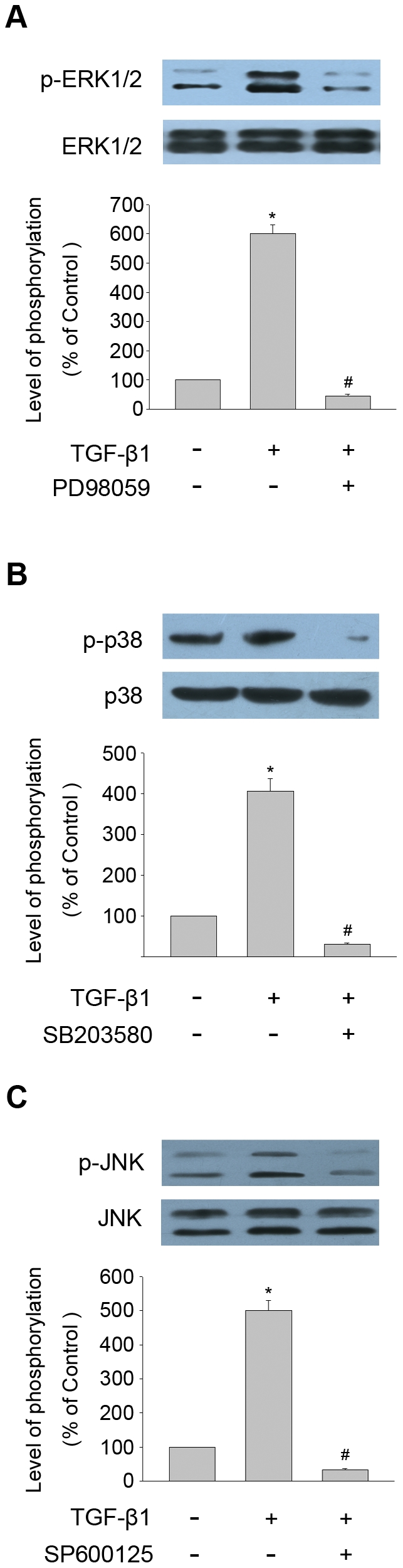
Inhibitory effect of PD98059, SB203580 or SP600125 on TGF-β1 induced MAPK pathways phosphorylation in THSF cells. THSF cells were pretreated with ERK inhibitor (PD98059, 30 µM), p38 inhibitor (SB203580, 10 µM) or JNK inhibitor (SP600125, 30 µM) for 1 h, respectively. Then the cells were subsequently treated with TGF-β1 (3 ng/ml) for 15 min (A) or 30 min (B, C), followed by protein extraction and Western blot analysis for total and phosphorylated ERK1/2 (A), p38 (B), and JNK (C). Data are representative of three independent experiments. *, P<0.05 vs. control; #, P<0.05 vs. TGF-β1 group.

The inhibitory effects of the three MAPK pathways-specific inhibitors on TGF-β1 induced MAPK phosphorylation were evaluated. THSF cells were pretreated with ERK inhibitor (PD98059, 30 µM), p38 inhibitor (SB203580, 10 µM) or JNK inhibitor (SP600125, 30 µM) for 1 h, respectively. Then the cells were stimulated with TGF-β1 (3 ng/ml) for 15 min (ERK) or 30 min (p38, JNK). The expressions of total and phosphorylated ERK1/2, p38, and JNK were determined by Western blot analysis. As shown in Figure 2, TGF-β1 induced phosphorylation of ERK, p38 or JNK were significantly inhibited by PD98059, SB203580 or SP600125, respectively.

### Effect of MAPK-specific inhibitors on expression and secretion of CTGF induced by TGF-β1

**Figure 3:**
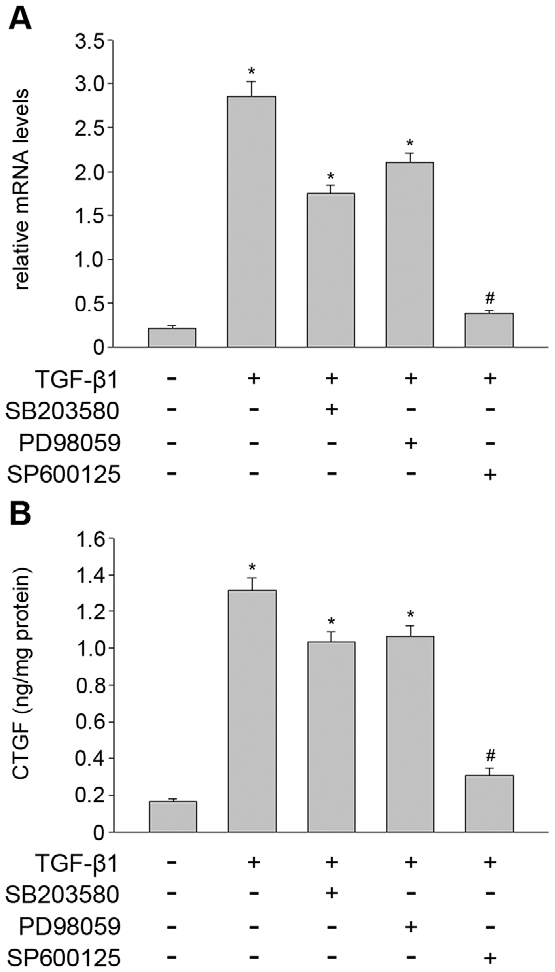
SP600125 inhibited TGF-β1 induced CTGF expression and secretion in THSF cells. THSF cells were pretreated with ERK inhibitor (PD98059, 30 µM), p38MAPK inhibitor (SB203580, 10 µM) or JNK inhibitor (SP600125, 30 µM) for 1 hour, respectively. Subsequently they were treated with TGF-β1 (3 ng/ml) for 24 hour. (A) CTGF mRNA expression levels were detected by real time PCR. (B) CTGF protein was measured in conditioned medium samples using ELISA and results were normalized for total protein concentration. Data are representative of tree independent experiments. *, P<0.05 vs. control; #, P<0.05 vs. TGF-β1 group.

To determine MAPK pathways requirements for the TGF-β1 induced CTGF expression, THSF cells were treated in the absence or presence of ERK inhibitor (PD98059, 30 µM), p38 inhibitor (SB203580, 10 µM) or JNK inhibitor (SP600125, 30 µM) for 1 h, respectively. TGF-β1 (3 ng/ml) was subsequently added to the culture for 24 h. Expression of CTGF mRNA was determined by real time PCR analysis. Figure 3 A shows that the presence of SP600125 markedly inhibited CTGF mRNA expression. In contrast, PD98059 and SB203580 showed weak effects on TGF-β1 induced CTGF mRNA expression.

In addition, the concentration of CTGF secretions into the medium was measured by ELISA analysis. As shown in Figure 3 B, compared with control, TGF-β1 significantly stimulated the secretions of CTGF after 24 h treatment. SP600125 markedly inhibited TGF-β1 stimulated CTGF secretion. However, SB203580 or PD98059 had no effect on the secretion of CTGF induced by TGF-β1.

### Effect of MAPK-specific inhibitors on expression of fibronectin and collagen I induced by TGF-β1

**Figure 4:**
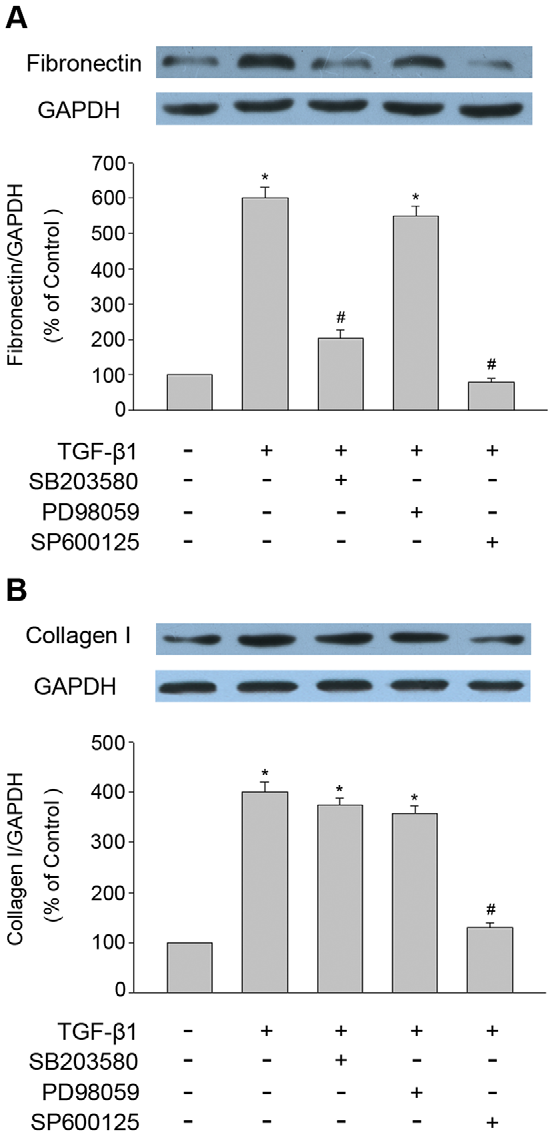
Effect of MAPK-specific inhibitors on expression of fibronectin and collagen I induced by TGF -β1 **in THSF cells.** THSF cells were pretreated with ERK inhibitor (PD98059, 30 µM), p38MAPK inhibitor (SB203580, 10 µM) or JNK inhibitor (SP600125, 30 µM) for 1 hour, respectively. Subsequently they were treated with TGF-β1 (3 ng/ml) for 24 hour. Expression of fibronectin and collagen I protein was determined by Western blot analysis. (A) SB203580 or SP600125 significant inhibited TGF-β1 induced fibronectin expression. (B) SP600125 significant suppressed TGF-β1 induced collagen I expression. Data are representative of three independent experiments. *, P<0.05 vs. control; **#**, P<0.05 vs. TGF-β1 group.

Next, we determined if MAPK pathways play any role in TGF-β1 induced fibronectin and collagen I expression. THSF cells were pretreated with ERK inhibitor (PD98059, 30 µM), p38MAPK inhibitor (SB203580, 10 µM) or JNK inhibitor (SP600125, 30 µM) for 1 hour, respectively. Subsequently they were treated with TGF-β1 (3 ng/ml) for 24 hour. Expression of fibronectin and collagen I protein was determined by Western blot analysis. As shown in Figure 4, TGF-β1 significantly upregulated expression of fibronectin and collagen I. Fibronectin expression was markedly decreased in the presence of SP600125 or SB203580. In contrast, no significant influence of PD98059 on fibronectin expression was observed. In addition, expression of collagen I was markedly attenuated by SP600125, whereas PD98059 or SB203580 showed weak effects on TGF-β1 induced collagen I expression.

### SP600125 inhibited JNK phosphorylation induced by penetrating corneal wound

**Figure 5:**
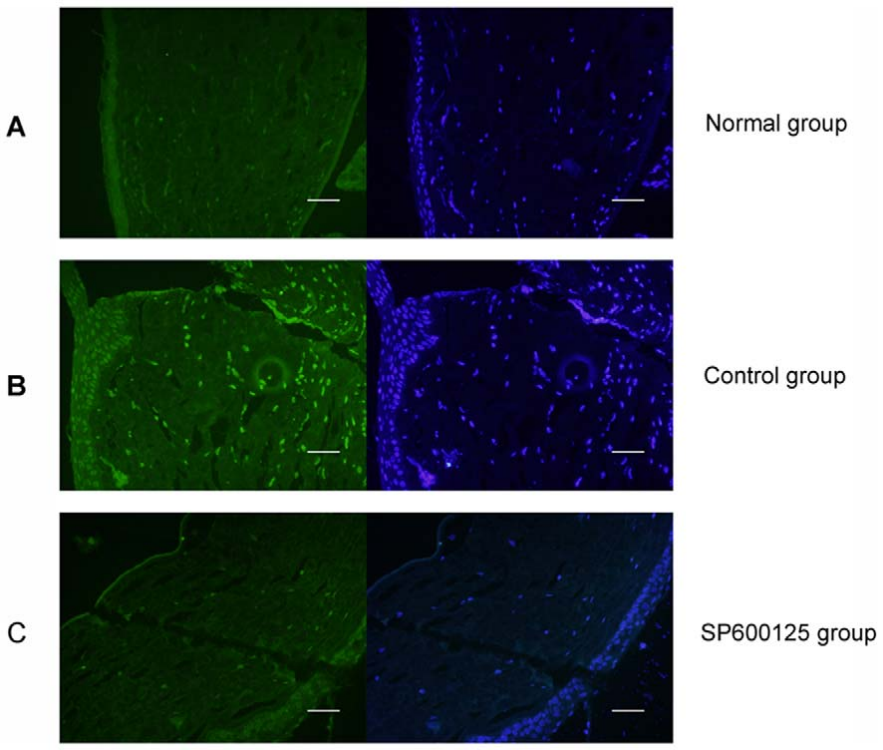
Evaluation of inhibitory effect of SP600125 on penetrating corneal wound induced JNK phosphorylation in Wistar rats. (A) p-JNK was examined by immunofluorescence analysis. Five micrometer corneal sections were stained with antibodies to p-JNK (green) as well as with nuclear staining dye (blue). There was little expression of P-JNK in the normal mice cornea of normal rats. (B) Penetrating injury was made in the central cornea of Wistar rats, control group received daily subconjunctival injection of physiological saline. Positive p-JNK staining was markedly increased in the corneal stroma at 1 d after injury. (C) Subconjunctival injection of SP600125 notably inhibited JNK activation in the corneal stroma at 1 d after injury. n = 4 rat in each group, Bars: 40 µm.

We next examined whether JNK was indeed phosphorylated in response to penetrating corneal wound and the effect of subconjunctival injection of SP600125 on JNK phosphorylation in vivo. Expression of p-JNK in the injured corneas was examined by immunofluorescence analysis. As shown in Figure 5 A, there was little expression of p-JNK in the cornea of normal rat, whereas positive p-JNK staining was markedly increased in the corneal stroma at 1 d after penetrating corneal wound (Figure 5 B). In SP600125 group, p-JNK expression was significantly reduced compared with control group received physiological saline treatment (Figure 5 C). These results suggest that JNK was activated after injury, subconjunctival injection of SP600125 notably inhibited JNK activation induced by penetrating corneal wound.

### SP600125 inhibited CTGF expression induced by penetrating corneal wound

**Figure 6:**
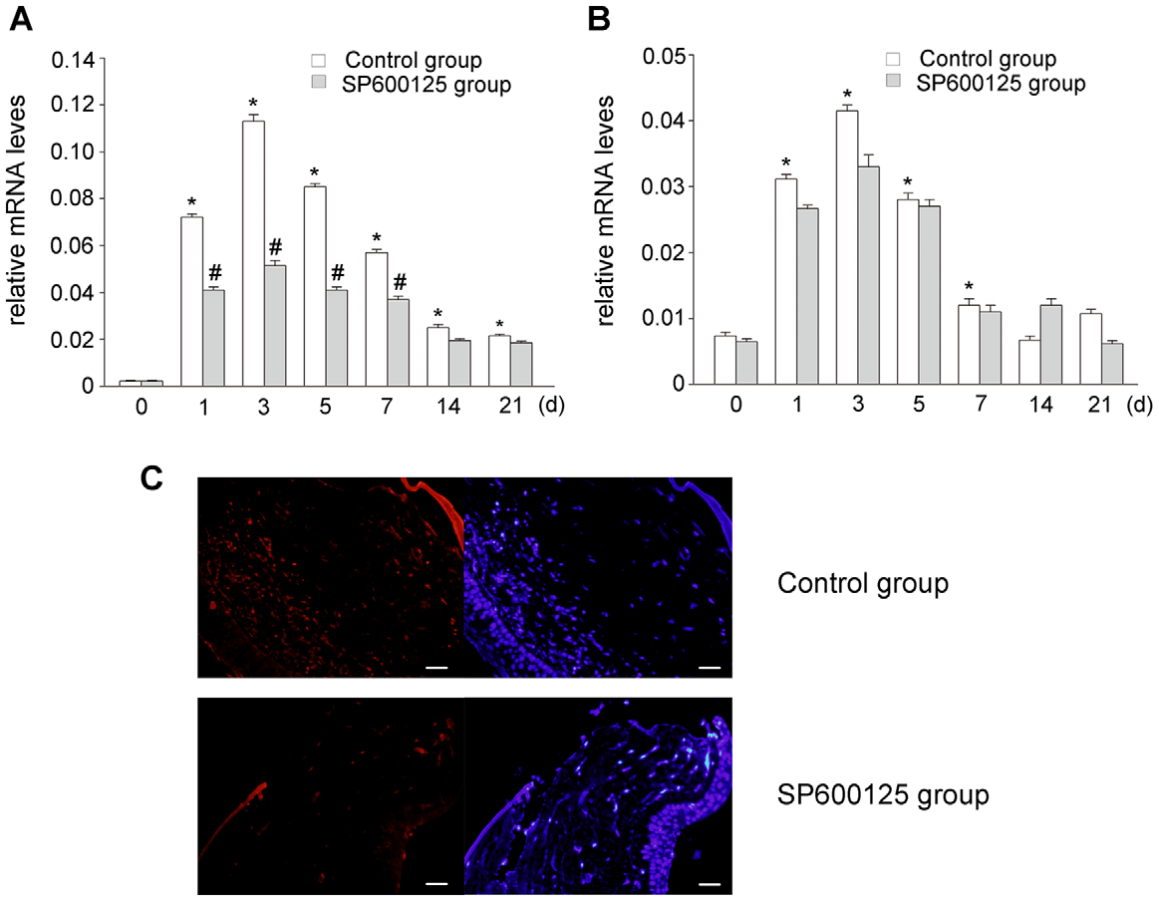
Evaluation of inhibitory effect of SP600125 on penetrating corneal wound induced CTGF expression in Wistar rats. (A) Real time PCR was used to measure CTGF mRNA expression. The expression of CTGF mRNA was upregulated significantly in wounded corneas and reached a peak at 3 d after injury, subconjunctival injection of SP600125 significantly inhibited injury-induced CTGF mRNA expression. (B) Real time PCR was used to measure TGF-β1 mRNA expression. Subconjunctival injection of SP600125 did not influence the expression of TGF-β1. Data are representative of three independent experiments. *, P<0.05 vs. 0 d; #, P<0.05 vs. control group at the same time point. (C) immunofluorescence was used to measure CTGF protein expression. Five micrometer corneal sections were stained with antibodies to CTGF (red) as well as with nuclear staining dye (blue). There was dramatic expression of CTGF protein in the corneal stroma at 3 d after injury. Subconjunctival injection of SP600125 notably inhibited CTGF expression. n = 4 rat in each group, Bars: 40 µm.

To investigate the effect of JNK on CTGF, TGF-β1 expression after corneal injury in vivo, JNK was inhibited with subconjunctival injection of SP600125. Expressions of CTGF, TGF-β1 mRNA were determined by real time PCR analysis and expression of CTGF protein was determined by immunofluorescence analysis. There was little expression of TGF-β1, CTGF mRNA in the corneal stroma without injury. After penetrating corneal wound, TGF-β1, CTGF mRNA expression markedly increased and reached a peak at 3 d. Inhibition of JNK with subconjunctival injection of SP600125, expression of CTGF mRNA was clearly reduced compared with control group received physiological saline treatment (Figure 6 A), but there was no change of TGF-β1 mRNA expression between groups (Figure 6 B). Figure 6 C shows that there was dramatic expression of CTGF protein in the corneal stroma at 3 d after injury. In SP600125 group, expression of CTGF protein was significantly reduced at 3 d after injury. These results suggest that inhibition of JNK with subconjunctival injection of SP600125 could inhibit CTGF expression in corneal wound healing, whereas it did not influence expression of TGF-β1.

### SP600125 inhibited corneal scarring in rat corneal wound healing

**Figure 7:**
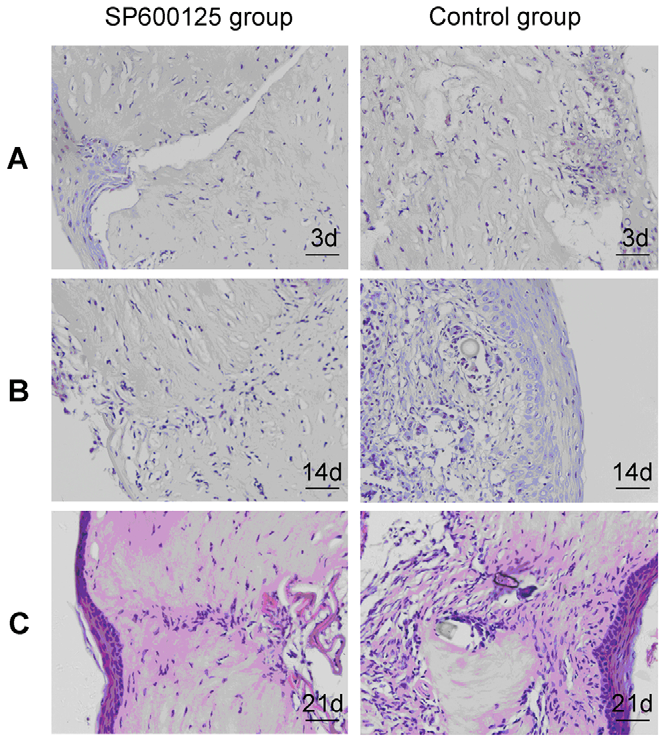
Evaluation of inhibitory effect of SP600125 on penetrating corneal wound induced corneal scarring in Wistar rats. A penetrating corneal wound model was created with Wistar rats and inhibition of JNK activation by subconjunctival injection of SP600125 daily post-wounding. (A) HE stained histological sections showed that corneal epithelial healing was almost complete at 3 d in both groups. Subconjunctival injection of SP600125 after injury daily markedly improved the architecture of cornea and reduced scarring and did not have a significant impact on wound stroma healing at 14 d (B), 21 d (C). n = 4 rat in each group, Bars: 40 µm.

Finally, whether inhibition of JNK activation could affect corneal scarring and corneal wound healing in vivo was investigated. HE stained histological sections showed that there were lamellar patterns and ordered collagen fibrils in normal Wistar rat corneas. As shown in Figure 7, corneal epithelial healing was almost completed at 3 d in both groups. In control group, the newly produced corneal stroma was comprised of disordered collagen fibrils and with loss of normal lamellar pattern. Whereas in SP600125 group, subconjunctival injection of SP600125 markedly improved the architecture of cornea and reduced scarring. In SP600125 group, corneal stroma healing did not completed at 3 d after injury, but subconjunctival injection of SP600125 post-wounding daily did not have a significant impact on wound stroma healing at 14 d and 21 d. These results suggest that exogenous addition of SP600125 inhibits corneal scarring in corneal wound healing.

## Discussion

The transparency of the cornea is very important for the maintenance of normal vision. Clinically, the major problem with corneal healing following injury or surgery is corneal scarring. A corneal scar may cause hypopsia or even blindness [29]. Fibroblast proliferation and matrix synthesis induced by growth factors have been assumed to be involved in initiating and maintaining fibrosis [30]. Although the underlying mechanisms are complex, many of the harmful aspects are mediated by the different effects of TGF-β1 as the final universal pathway. TGF-β1 has been implicated in many fibrotic disorders of the lung, liver, kidney and pancreas. Treatment with antisense oligonucleotides or antibodies to TGF-β1 in cell culture or animal models decreased extracellular matrixc (ECM) synthesis or reduced scarring. Many of the effect of TGF-β1 on ECM production, collagen synthesis and cell proliferationare mediated by CTGF. Namely, CTGF plays a critical role in mediating the fibroproliferative effects of TGF-β1. Levels of CTGF are correlated with increased expression of ECM, such as collagen I, integrins, and fibronectin. Therefore, it is important to define the signaling pathway through which TGF-β1 induces CTGF expression.

It is widely accepted that TGF-β1 stimulation results in the activation of the MAPK pathways [27], [31]. The MAPK pathways are a family of serine threonine protein kinases that are activated in response to a variety of extracellular stimuli [32]. ERK, p38 and JNK constitute three major subfamilies of MAPK [33]. ERK plays a major role in cell proliferation and differentiation, as well as in survival mediated by various growth factors. JNK and p38 are activated by various inflammatory cytokines and environmental stressors and they play important roles in apoptosis and cytokine production. Studies in renal fibroblasts and mesangial cells demonstrated the requirement of ERK for TGF-β1 induced CTGF expression [34], [35]. However, in smooth muscle cells both ERK and JNK are required for CTGF induction by TGF-β1 [36]. In another study using lung fibroblasts, it was determined that CTGF expression was dependent on JNK, not p38 or ERK [25]. Inhibition of JNK suppressed TGF-β1 induced CTGF and collagen I expression in mesangial cells [37]. In cultures of human corneal epithelial cells, synthesis of CTGF induced by TGF-β1 is through ERK [38]. Studies have shown that there are differences in the requirement of specific MAPK for CTGF expression inducted by TGF-β1 and this discrepancy may be explained due to different cell lines and species.

In our study, THSF cells stimulated with TGF-β1 induced a rapid activation of ERK, p38 and JNK (Figure 1). Pretreatment of THSF cells with three MAPK pathways-specific inhibitors (PD98059, SB203580 or SP600125) could significantly inhibited the activation of ERK, p38 or JNK, respectively (Figure 2). To elucidate which member of MAPK may be responsible for the TGF-β1 induced CTGF, fibronectin and collagen I expression in THSF cells, activation of p38, ERK and JNK were inhibited by incubating THSF cells with SB203580, PD98059 and SP600125 for 1 hour before stimulation with TGF-β1, 24 h later expression of CTGF, fibronectin and collagen I were determined. Our data showed that inhibition of JNK by SP600125 suppressed expression of CTGF, fibronectin and collagen I in response to TGF-β1 stimulation, whereas inhibition of p38 by SB203580 only resulted in suppression of TGF-β1 induced fibronectin expression. On the other hand, inhibition of ERK by PD98059 did not significantly alter expression of CTGF, fibronectin or collagen I in response to TGF-β1 (Figure 3 A, 4). CTGF is a secreted protein. We also measured the concentrations of CTGF in cell culture supernatants. Our data showed that TGF-β1 significantly increased CTGF secretions and SP600125 markedly inhibited TGF-β1 stimulated CTGF secretion. However, SB203580 or PD98059 had no effect on the secretion of CTGF induced by TGF-β1 (Figure 3 B). These findings indicate that JNK is a key pathway in modulating the signals through which TGF-β1 promotes CTGF, fibronectin and collagen I expression in corneal fibroblasts. Previous studies demonstrated that inhibition of JNK can effectively inhibit TGF-β1 induced CTGF expression in corneal fibroblasts [15], [16]. The present results concur with the previous report and expand the findings by demonstrating that p38 and ERK are not required for CTGF induction by TGF-β1.

Our group previously demonstrated that TGF-β1 and CTGF were upregulated dramatically in corneal stroma during corneal wound healing, CTGF expression decreased dramatically in group that wounded eyes were injected with TGF-β1 antibody subconjunctivaly. TGF-β1 neutralizing antibody may inhibit the biological functions of TGF-β1. Therefore, we indicated that TGF-β1 could induce CTGF expression in vivo. To further investigate the role of JNK in mediating CTGF expression and corneal scarring in vivo, a penetrating corneal wound model was created and JNK was inhibited with subconjunctival injection of SP600125. Immunofluorescence results showed that there was little expression of p-JNK in corneas of normal rat, but p-JNK expression was greatly increased in the corneal stroma after penetrating corneal wound. Subconjunctival injection of SP600125 could inhibite p-JNK expression compared with control group received physiological saline treatment (Figure 5). This indicated that subconjunctival injection of SP600125 could significantly inhibit activation of JNK induced by corneal wounding. It was also found that expression of TGF-β1 mRNA, CTGF mRNA and protein markedly increased in corneal stroma after injury. Subconjunctival injection of SP600125 could clearly inhibit CTGF mRNA and protein expression, but did not influence TGF-β1 mRNA expression (Figure 6). These results suggest that inhibition of JNK with subconjunctival injection of SP600125 could inhibit CTGF expression in corneal wound healing. Histological results showed that the newly produced corneal stroma was comprised of disordered collagen fibrils and with loss of normal lamellar pattern in control group, whereas subconjunctival injection of SP600125 markedly improved the architecture of corneal stroma and reduced corneal scarring (Figure 7). The present results indicated that inhibition of JNK could significantly inhibit corneal scarring after injury. These findings established that excessive CTGF expression was responsible for corneal scarring, and inhibition of JNK could markedly decrease excessive expression of CTGF, and down-regulation of CTGF expression caused a reduction of corneal scarring.

It was also found that corneal epithelial healing was almost complete at 3 d after injury in both groups and subconjunctival injection of SP600125 did not have a significant impact on wound stroma healing at 14 d and 21 d. Inhibition of JNK could effectively reduce corneal scarring without having a deleterious effect on healing in vivo. Previous reports have indicated that CTGF cooperates with fibronectin in enhancing the attachment and migration of human corneal epithelial cells [39]. Also, recent studies demonstrated that in cultures of human corneal epithelial cells, TGF-β1 induced CTGF synthesis through ERK and this is required for cell migration [38]. However, it has been shown that during re-epithelialisation of mouse corneas, TGF-β1 was found to enhance re-epithelialisation by enhancing cell migration via p38 [40].

In summary, the present study demonstrates that TGF-β1 and penetrating corneal wound induce JNK activation, and JNK mediates CTGF expression induced by TGF-β1 and penetrating corneal wound. Inhibition of JNK could inhibit excessive expression of CTGF and subsequent corneal scarring without clearly affecting wound healing in vivo. JNK could potentially serve as a new strategy to reduce corneal scar formation.

## Materials and Methods

### Animals

Wistar rats (10-week-old male, 200–300 g) were obtained from the Animal Supplier Center of Shandong University. All the animal studies were approved by the Ethics Committee of Shandong University, and animals were used in compliance with the Association for Research in Vision and Ophthalmology Statement for the Use of Animals in Ophthalmic and Vision Research.

### Reagents

TGF-β1 was obtained from Peprotech (Rocky Hill, NJ). Anti-Collagen I antibody was purchased from Abcam (Cambridge, MA), Antibodies against CTGF, fibronectin were purchased from Santa Cruz Biotechnology, Inc. (Santa Cruz, CA). Antibodies against JNK, ERK1/2, p38 MAPK, phospho-JNK (Thr183/Tyr185), phospho-ERK1/2 (Thr202/Tyr204) and phospho-p38 MAPK (Thr180/Tyr182) were obtained from Cell Signaling Technology, Inc. (Danvers, MA). PD98059 and SB203580 were purchased from Calbiochem (San Diego, CA), SP600125 was obtained from A. G. Scientific, Inc (San Diego, CA).

### Cell culture and treatment

THSF cells were maintained in Dulbecco Modified Eagle Medium (DMEM; Invitrogen Life Technologies, Carlsbad, CA) with 10% fetal bovine serum (Gibco, Carlsbad, CA) in a humidified 5% CO^2^ incubator at 37°C. The cells were seeded into 6-well plates at a density of 2×10^5^ cells per well in normal growth medium. Before treatment, the cells were cultured in serum-free DMEM for 24 h. The cells were treated in the absence or presence of ERK inhibitor (PD98059, 30 µM), p38 inhibitor (SB203580, 10 µM) or JNK inhibitor (SP600125, 30 µM) for 1 h, respectively; TGF-β1 (3 ng/ml) was subsequently added to the culture for the determined time depending on the different purposes. The cells of control group were added to an equal volume of serum-free medium. The culture media and cells were harvested at the indicated time-points for measurement of RNA and protein levels.

### Enzyme-Linked Immunosorbent Assay (ELISA)

CTGF is a secreted protein, we measured the concentrations of CTGF in cell culture supernatants by ELISA according to the manufacturer's instructions (Uscn Life Science Inc.,Wuhan, China). Standards were run with each assay to ensure accuracy. For quantitative results, the signal of unknown samples was compared against a standard curve. CTGF levels were normalized for total protein content in the sample using BCA Protein Assay Kit (Beyotime, Jiangsu, China) and were expressed as ng mg^−1^ protein for three replicate samples for each condition.

### Animal model

The previously described experimental model was used for this study [41]. Briefly, Wistar rats were anesthetized with chloral hydrate and placed beneath a stereoscopic microscope. After instilling Oxybuprocaine Hydrochloride eye drops for local anesthesia, a penetrating linear incision of uniform size (3 mm) was made with a scalpel in the center of cornea and treated with interrupted suture. Erythromycin ophthalmic ointment was applied for prevention of infection. Only one eye of each animal was operated, another eye was used as the control. All corneal surgeries were performed by the same surgeon to ensure consistency across specimens. The process of corneal wound healing was observed everyday by slit lamp. Only those corneas that showed clinically normal healing without complication were used in this study.

For experimental group, the wounded eyes received subconjunctival injection of SP600125 (50 µM) daily after operation. While control group, the wounded eyes received subconjunctival injection of physiological saline. The eyes of rats were examined daily by slit lamp and sacrificed at 1, 3, 5, 7, 14 and 21 days following the treatment.

### HE and Immunofluorescent staining

Histological analysis of the cornea was as previously described [42]. Briefly, half corneas of rats were fixed in 3.7% formaldehyde for 24 hours and then were frozen in an optimal cutting temperature (OCT; Sakura Finetek, Torrance, CA) compound. Five micrometer corneal sections were sliced with a cryostat. Parts of the sections were stained with hematoxylin and eosin. Sections for immunofluorescence analysis were blocked with 2% BSA in PBS, and primary antibodies were applied overnight in a moist chamber at 4°C. Fluorescein conjugated secondary antibodies was applied for 1 hour in a dark incubation chamber at room temperature. The negative control was prepared by incubation with secondary antibody alone. HE stain was observed and photographed using a Nikon UFX-IIA microscope, immunofluorescence stain was examined under a fluorescence microscope. Every sample was treated simultaneously to reduce variations among fixation, embedding and section procedures.

### Real-time RT-PCR

Real time RT-PCR was performed as previously reported method [43]. In brief, RNA was isolated with Trizol, 2 µg RNA was reverse-transcribed using oligo (dT), random hexamers and Moloney murine leukemia virus (MMLV) reverse transcriptase (Promega) in a final volume of 20 µl. The resulting cDNA was used for quantitative real-time PCR with SYBR Green I (Tiangen Biotech, Beijing, China) on ABI 7000 (Applied Biosystem Inc., CA, USA). Primers sequences were: CTGF: forward 5′-GCTGGAGAAGCAGAGTCGTC-3′, reverse 5′-CCACAGAACTTAGCCCGG TA-3′; TGF-β1: forward 5′- GTCAACTGTGGAGCAACACG-3′, reverse 5′-AGAC AGCCACTCAGGCGTA-3′; β-actin: forward 5′-CGTTGACATCCGTAAAGACC-3′, reverse 5′-TAGAGCCACC AATCCACA-3′. All real-time PCR reactions for each cytokine were performed in triplicate. Gene expression levels were calculated and normalized by dividing the calculated values for the mRNA samples by that of β-actin mRNA at the same time point.

### Western blot analysis

Western blotting proceeded as previously described [44]. Briefly, cultured cells were collected at the indicated times and lysed by shaking at 4°C for 30 min in RIPA buffer (50 mM Tris-HCl, 1% NP-40, 0.25% Na-deoxycholate, and 150 mM NaCl, 1 mM Na_3_VO_4_ and NaF) containing protease inhibitors (1 µg/ml each of EDTA and phenylmethylsulfonyl fluoride). Cell lysates were centrifuged at 12,000 g for 15 min at 4°C. The supernatant was transferred to new Eppendorf tubes (Zhizhuang Biotech, Co., Ltd., Shanghai, China) and boiled for 5 min in sample buffer (12 mM Tris-HCl, 10% glycerol, 10% sodium dodecyl sulfate and 1% 2-mercaptoethanol and 0.1% bromophenol blue, pH 6.8). Total protein was quantified and 30 µg protein samples were subjected to 10% sodium dodecyl sulfate-polyacrylamide gel electrophoresis, and then transferred to nitrocellulose membranes. The membranes were blocked with 5% skim milk in Tris-buffered saline containing 0.05% Tween-20 for 2 h at room temperature before overnight incubation at 4°C with primary antibodies. After incubation with primary antibodies, nitrocellulose membranes were extensively washed with Tris-buffered saline with 0.05% Tween-20 and incubated with secondary antibodies for 2 h at 37°C. Protein bands were visualized using enhanced chemiluminescence as described by the supplier (GE Healthcare). Densitometric analysis has been carried out with Quantity One software (Bio-Rad, Hercules, CA).

### Statistical analysis

Results were expressed as means ± SD. Student's t-test was used to compare two groups of Results, whereas ANOVA was used in multiple group comparisons. P<0.05 was considered statistically significant. Data analysis was carried out with the Statistical Package for Social Sciences (SPSS version 11.0).

## References

[1] Limburg H, Barria von Bischhoffshausen F, Gomez P, Silva JC, Foster A (2008). Review of recent surveys on blindness and visual impairment in Latin America.. Br J Ophthalmol.

[2] Fukada T, Civic N, Furuichi T, Shimoda S, Mishima K (2008). The zinc transporter SLC39A13/ZIP13 is required for connective tissue development; its involvement in BMP/TGF-beta signaling pathways.. PLoS One.

[3] Dessein A, Chevillard C, Arnaud V, Hou XY, Hamdoun AA (2009). Variants of CTGF are associated with hepatic fibrosis in Chinese, Sudanese, and Brazilians infected with Schistosomes.. Journal of Experimental Medicine.

[4] Kuiper EJ, Van Nieuwenhoven FA, de Smet MD, van Meurs JC, Tanck MW (2008). The Angio-Fibrotic Switch of VEGF and CTGF in Proliferative Diabetic Retinopathy.. PLoS One.

[5] Qi W, Chen X, Polhill TS, Sumual S, Twigg S (2006). TGF-beta1 induces IL-8 and MCP-1 through a connective tissue growth factor-independent pathway.. Am J Physiol Renal Physiol.

[6] Song JJ, Aswad R, Kanaan RA, Rico MC, Owen TA (2007). Connective tissue growth factor (CTGF) acts as a downstream mediator of TGF-beta1 to induce mesenchymal cell condensation.. J Cell Physiol.

[7] Chujo S, Shirasaki F, Kawara S, Inagaki Y, Kinbara T (2005). Connective tissue growth factor causes persistent proalpha2(I) collagen gene expression induced by transforming growth factor-beta in a mouse fibrosis model.. J Cell Physiol.

[8] Guo F, Carter DE, Leask A (2011). Mechanical tension increases CCN2/CTGF expression and proliferation in gingival fibroblasts via a TGFbeta-dependent mechanism.. PLoS One.

[9] Phanish MK, Winn SK, Dockrell ME (2010). Connective tissue growth factor-(CTGF, CCN2)–a marker, mediator and therapeutic target for renal fibrosis.. Nephron Exp Nephrol.

[10] Chen CC, Lau LF (2009). Functions and mechanisms of action of CCN matricellular proteins.. Int J Biochem Cell Biol.

[11] Shi Wen X, Leask A, Abraham D (2008). Regulation and function of connective tissue growth factor/CCN2 in tissue repair, scarring and fibrosis.. Cytokine Growth Factor Rev.

[12] Garrett Q, Khaw PT, Blalock TD, Schultz GS, Grotendorst GR (2004). Involvement of CTGF in TGF-beta1-stimulation of myofibroblast differentiation and collagen matrix contraction in the presence of mechanical stress.. Invest Ophthalmol Vis Sci.

[13] Blalock TD, Duncan MR, Varela JC, Goldstein MH, Tuli SS (2003). Connective tissue growth factor expression and action in human corneal fibroblast cultures and rat corneas after photorefractive keratectomy.. Invest Ophthalmol Vis Sci.

[14] Wu XY, Yang YM, Guo H, Chang Y (2006). The role of connective tissue growth factor, transforming growth factor beta1 and Smad signaling pathway in cornea wound healing.. Chin Med J (Engl).

[15] Chang Y, Wu XY (2009). The role of c-Jun N-terminal kinases 1/2 in transforming growth factor beta(1)-induced expression of connective tissue growth factor and scar formation in the cornea.. J Int Med Res.

[16] Chang Y, Wu XY (2010). JNK1/2 siRNA inhibits transforming-growth factor-beta1-induced connective tissue growth factor expression and fibrotic function in THSFs.. Mol Cell Biochem.

[17] Daniels JT, Schultz GS, Blalock TD, Garrett Q, Grotendorst GR (2003). Mediation of transforming growth factor-beta(1)-stimulated matrix contraction by fibroblasts: a role for connective tissue growth factor in contractile scarring.. Am J Pathol.

[18] Blalock TD, Yuan R, Lewin AS, Schultz GS (2004). Hammerhead ribozyme targeting connective tissue growth factor mRNA blocks transforming growth factor-beta mediated cell proliferation.. Exp Eye Res.

[19] Zi Z, Klipp E (2007). Constraint-Based Modeling and Kinetic Analysis of the Smad Dependent TGF-beta Signaling Pathway.. PLoS One.

[20] Peterziel H, Unsicker K, Krieglstein K (2002). TGFbeta induces GDNF responsiveness in neurons by recruitment of GFRalpha1 to the plasma membrane.. J Cell Biol.

[21] Ge C, Xiao G, Jiang D, Franceschi RT (2007). Critical role of the extracellular signal-regulated kinase-MAPK pathway in osteoblast differentiation and skeletal development.. J Cell Biol.

[22] Imamichi Y, Waidmann O, Hein R, Eleftheriou P, Giehl K (2005). TGF beta-induced focal complex formation in epithelial cells is mediated by activated ERK and JNK MAP kinases and is independent of Smad4.. Biol Chem.

[23] Lien SC, Usami S, Chien S, Chiu JJ (2006). Phosphatidylinositol 3-kinase/Akt pathway is involved in transforming growth factor-beta1-induced phenotypic modulation of 10T1/2 cells to smooth muscle cells.. Cell Signal.

[24] Suk FM, Chen CH, Lin SY, Cheng CJ, Yen SJ (2009). 15-deoxy-Delta(12,14)-prostaglandin J(2) inhibits fibrogenic response in human hepatoma cells.. Toxicol Lett.

[25] Utsugi M, Dobashi K, Ishizuka T, Masubuchi K, Shimizu Y (2003). C-Jun-NH2-terminal kinase mediates expression of connective tissue growth factor induced by transforming growth factor-beta1 in human lung fibroblasts.. Am J Respir Cell Mol Biol.

[26] Black SA, Palamakumbura AH, Stan M, Trackman PC (2007). Tissue-specific mechanisms for CCN2/CTGF persistence in fibrotic gingiva: interactions between cAMP and MAPK signaling pathways, and prostaglandin E2-EP3 receptor mediated activation of the c-JUN N-terminal kinase.. J Biol Chem.

[27] Saika S, Ikeda K, Yamanaka O, Miyamoto T, Ohnishi Y (2005). Expression of Smad7 in mouse eyes accelerates healing of corneal tissue after exposure to alkali.. Am J Pathol.

[28] Yamanaka O, Saika S, Ohnishi Y, Kim Mitsuyama S, Kamaraju AK (2007). Inhibition of p38MAP kinase suppresses fibrogenic reaction in conjunctiva in mice.. Mol Vis.

[29] Cursiefen C (2007). Immune privilege and angiogenic privilege of the cornea.. Chem Immunol Allergy.

[30] Munireddy S, Kavalukas SL, Barbul A (2010). Intra-abdominal healing: gastrointestinal tract and adhesions.. Surg Clin North Am.

[31] Wang XM, Zhang YZ, Kim HP, Zhou ZH, Feghali Bostwick CA (2006). Caveolin-1: a critical regulator of lung fibrosis in idiopathic pulmonary fibrosis.. Journal of Experimental Medicine.

[32] Zhang J, Bian HJ, Li XX, Liu XB, Sun JP (2010). ERK-MAPK signaling opposes rho-kinase to reduce cardiomyocyte apoptosis in heart ischemic preconditioning.. Mol Med.

[33] Bogoyevitch MA, Ngoei KR, Zhao TT, Yeap YY, Ng DC (2010). c-Jun N-terminal kinase (JNK) signaling: recent advances and challenges.. Biochim Biophys Acta.

[34] Chen Y, Blom IE, Sa S, Goldschmeding R, Abraham DJ (2002). CTGF expression in mesangial cells: involvement of SMADs, MAP kinase, and PKC.. Kidney Int.

[35] Leivonen SK, Hakkinen L, Liu D, Kahari VM (2005). Smad3 and extracellular signal-regulated kinase 1/2 coordinately mediate transforming growth factor-beta-induced expression of connective tissue growth factor in human fibroblasts.. J Invest Dermatol.

[36] Xie S, Sukkar MB, Issa R, Oltmanns U, Nicholson AG (2005). Regulation of TGF-beta 1-induced connective tissue growth factor expression in airway smooth muscle cells.. Am J Physiol Lung Cell Mol Physiol.

[37] Sohn M, Tan Y, Klein RL, Jaffa AA (2005). Evidence for low-density lipoprotein-induced expression of connective tissue growth factor in mesangial cells.. Kidney Int.

[38] Secker GA, Shortt AJ, Sampson E, Schwarz QP, Schultz GS (2008). TGFbeta stimulated re-epithelialisation is regulated by CTGF and Ras/MEK/ERK signalling.. Exp Cell Res.

[39] Sugioka K, Yoshida K, Kodama A, Mishima H, Abe K (2010). Connective tissue growth factor cooperates with fibronectin in enhancing attachment and migration of corneal epithelial cells.. Tohoku J Exp Med.

[40] Saika S, Okada Y, Miyamoto T, Yamanaka O, Ohnishi Y (2004). Role of p38 MAP kinase in regulation of cell migration and proliferation in healing corneal epithelium.. Invest Ophthalmol Vis Sci.

[41] Matsuda A, Tagawa Y, Matsuda H, Nishihira J (1997). Expression of macrophage migration inhibitory factor in corneal wound healing in rats.. Invest Ophthalmol Vis Sci.

[42] Pang K, Du L, Wu X (2010). A rabbit anterior cornea replacement derived from acellular porcine cornea matrix, epithelial cells and keratocytes.. Biomaterials.

[43] Guo H, Wu X, Yu FS, Zhao J (2008). Toll-like receptor 2 mediates the induction of IL-10 in corneal fibroblasts in response to Fusarium solu.. Immunol Cell Biol.

[44] Ren M, Gao L, Wu X (2010). TLR4: the receptor bridging Acanthamoeba challenge and intracellular inflammatory responses in human corneal cell lines.. Immunol Cell Biol.

